# Acceleration of vessel‐selective dynamic MR Angiography by pseudocontinuous arterial spin labeling in combination with Acquisition of ConTRol and labEled images in the Same Shot (ACTRESS)

**DOI:** 10.1002/mrm.27619

**Published:** 2018-12-02

**Authors:** Yuriko Suzuki, Thomas W. Okell, Noriyuki Fujima, Matthias J.P. van Osch

**Affiliations:** ^1^ Institute of Biomedical Engineering University of Oxford Oxford United Kingdom; ^2^ C.J. Gorter Center for High Field MRI, Department of Radiology Leiden University Medical Center Leiden The Netherlands; ^3^ Wellcome Centre for Integrative Neuroimaging, FMRIB, Nuffield Department of Clinical Neurosciences University of Oxford Oxford United Kingdom; ^4^ Department of Diagnostic and Interventional Radiology Hokkaido University Hospital Hokkaido Japan

**Keywords:** arterial spin labeling, dynamic MR angiography, noncontrast MRA, vessel‐selective pCASL

## Abstract

**Purpose:**

The recently introduced “Acquisition of ConTRol and labEled imaging in the Same Shot” (ACTRESS) approach was designed to halve the scan time of arterial spin labeling (ASL) ‐based 4D‐MRA by obtaining both labeled and control images in a single Look‐Locker readout. However, application for vessel‐selective labeling remains difficult. The aim of this study was to achieve a combination of ACTRESS and vessel‐selective labeling to halve the scan time of vessel‐selective 4D‐MRA.

**Methods:**

By Bloch equation simulations, Look‐Locker pseudocontinuous‐ASL (pCASL) was optimized to achieve constant static tissue signal across the multidelay readout, which is essential for the ACTRESS approach. Additionally, a new subtraction scheme was proposed to achieve visualization of the inflow phase even when labeled blood will have already arrived in the distal arteries during the first phase acquisition due to the long duration of the pCASL labeling module. In vivo studies were performed to investigate the signal variation of the static tissue, as well as to assess image quality of vessel‐selective 4D‐MRA with ACTRESS.

**Results:**

In in vivo studies, the mean signal variation of the static tissue was 8.98% over the Look‐Locker phases, thereby minimizing the elevation of background signal. This allowed visualization of peripheral arteries and slowly arriving arterial blood with image quality as good as conventional pCASL within half the acquisition time. Vessel‐selective pCASL‐ACTRESS enabled the separated visualization of vessels arising from internal and external carotid arteries within this shortened acquisition time.

**Conclusion:**

By combining vessel‐selective pCASL and ACTRESS approach, 4D‐MRA of a single targeted arterial tree was achieved in a few minutes.

## 1 INTRODUCTION

Dynamic MRA (4D‐MRA) using arterial spin labeling (ASL) provides several advantages over contrast‐enhanced (CE) 4D‐MRA in the brain. For example, vessel‐selective imaging that exclusively visualizes the downstream arterial tree of an individually targeted artery, can be achieved by applying spatially‐selective labeling pulses. Several previous studies report the usefulness of such vessel‐selective MRA, for example, for detecting the feeding arteries of an AVM.^1^, ^2^ Also, ASL has a higher flexibility to achieve both high temporal and spatial resolution, because it is not limited to capture all necessary information during the first passage of a bolus of contrast agent. Therefore, ASL data acquisition can be repeated until sufficient information is acquired to achieve the desired spatial and temporal resolution. The scan time of ASL‐based 4D‐MRA will, however, be generally much longer than CE‐4D‐MRA, especially because ASL techniques require the acquisition of 2 image types, i.e., labeling and control conditions, which are generally acquired as separate acquisitions to subtract out the static tissue signal and isolate the ASL signal.

The recently proposed approach “Acquisition of ConTRol and labEled imaging in the Same Shot” (ACTRESS) was designed to shorten the scan time of non–vessel‐selective ASL‐based 4D‐MRA by nearly a factor of 2.^3^ In this approach, a single control image is acquired before applying the labeling pulse, which is followed by Look‐Locker readouts^4^ at multiple inversion‐times (TI) after the labeling pulse (Figure 1 in Suzuki et al),^3^ which provide the labeled images. The subtraction is performed between all labeled images (2nd – Nth phase) and the single control image, that is, the first phase acquired before the labeling pulse.

**Figure 1:**
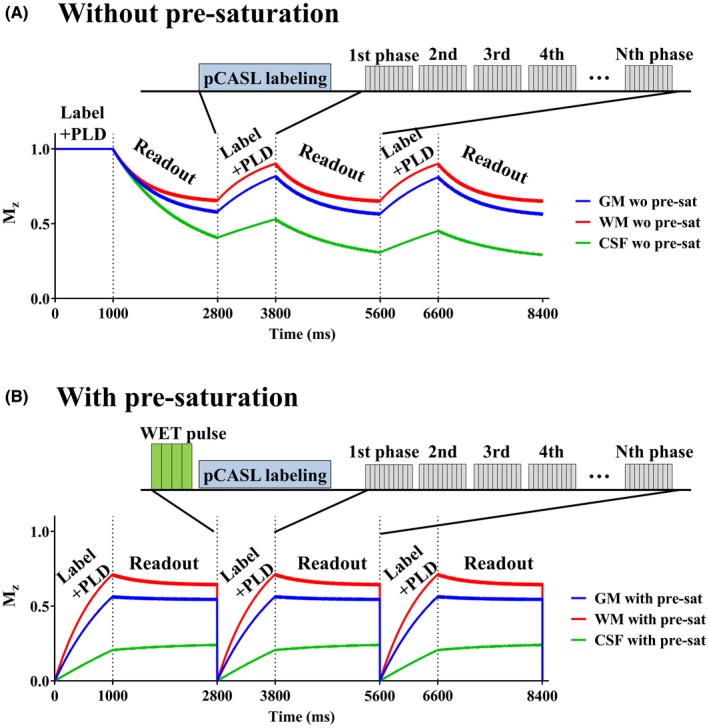
Simulated longitudinal magnetization (M_z_) of the background static tissue as obtained by Bloch equation simulations. A, Without a presaturation pulse before the pCASL labeling train, M_z_ varies considerably during Look‐Locker multi‐PLD readout. B, By applying a presaturation pulse before the pCASL labeling train, variation of M_z_ during the readout becomes much smaller

For this ACTRESS approach, one of the most important requirements to obtain similar image quality to conventional ASL‐based 4D‐MRA is to keep the signal intensity of the static tissue constant over all multi‐TI readouts. This ensures that the signal from the static tissue will be effectively eliminated by subtraction. Variation of the static tissue signal over different TIs would result in elevated background signal after subtraction, which would weaken the contrast of the smaller peripheral arteries, especially in later phases. In the ACTRESS approach, constant signal over all different TIs is achieved by 2 essential elements of the sequence design: (i) the readout interval is kept constant to establish a steady‐state of static tissue, and (ii) the effect of the labeling pulse (and any other prepulses) on the magnetization of the imaging volume is minimized, which would otherwise introduce unwanted signal changes over the Look‐Locker readout.

The ACTRESS approach was optimized based on the pulsed ASL (PASL) technique. When considering this approach for vessel‐selective imaging, however, some difficulties are encountered. First, for vessel‐selective labeling by PASL a spatially‐selective labeling slab is applied parallel to the target artery in the inferior‐superior direction, i.e., as an approximately coronal or sagittal slab so that a large volume of the targeted arterial blood is labeled.^5^ Such a coronal/sagittal labeling slab will be perpendicular to the transverse imaging slices and the intersection of the labeling slab and the imaging volume will directly violate the above‐mentioned second design element of the ACTRESS sequence. Pseudocontinuous ASL (pCASL) could, therefore, be an attractive option for the labeling module, because in pCASL the labeling of arterial blood is achieved by flow‐driven pseudoadiabatic inversion within a thin labeling plane perpendicular to the flow direction, that is, parallel to the imaging volume. A spatially varying pattern as needed for vessel‐selective ASL can be achieved by applying additional in‐plane gradients,^6^ while keeping the same parallel geometry of the labeling plane to the imaging volume. The use of pCASL labeling does, therefore, adhere to the second design‐element. However, it violates the first design element of the ACTRESS approach: in pCASL a long train of labeling pulses is needed to generate enough labeled arterial blood. For the magnetization in the imaging volume, however, such a long period of labeling will act like a long recovery period, free from disturbances by RF pulses, thereby destroying the steady‐state condition and introducing large signal changes of the static tissue during the Look‐Locker readout.

In this article, we present how the ACTRESS approach can be combined with vessel‐selective pCASL to achieve a fast acquisition sequence for vessel‐selective 4D‐MRA.

## 2 METHODS

### 2.1 Optimization of the static tissue signal

The concept for pCASL‐ACTRESS is as follows. In pCASL large signal variations of the static tissue will occur over the Look‐Locker readout due to longitudinal relaxation during the long pCASL labeling period, which will destroy the steady‐state condition of the static tissue (Figure 1A). To minimize this signal variation, a presaturation pulse is applied before the labeling module (Figure 1B). The transition to the steady‐state of the static tissue signal will subsequently be influenced by several parameters, such as the duration of the labeling train, the postlabeling delay (PLD), the number of excitation pulses per shot, their flip angle (FA), and TR. Optimal behavior can be designed by means of Bloch equation simulation, although other criteria, such as the desired spatial and/or temporal resolution and the labeling duration, put additional constraints on these parameters. Bloch equation simulation was performed, and a parameter setting that produces stable longitudinal magnetization behavior over Look‐Locker readout was determined (see the In vivo healthy volunteer study section below). The T_1_ of gray matter (GM), white matter (WM), and cerebrospinal fluid (CSF) were assumed to be 1200, 800, and 4300 ms, respectively.^7^, ^8^, ^9^

As input for the simulations, the readout‐module should be defined. For this study, 3D turbo field EPI (TFEPI) was chosen as the readout‐module, in which a segmented EPI readout is performed in between the excitation pulses of the TFE sequence. The number of k‐lines acquired per EPI‐train is referred to as “EPI‐factor”. The use of EPI helps to achieve fast acquisition as needed for the desired high temporal resolution, as well as to reduce the number of RF excitation pulses, which reduces the saturation of ASL signal and helps, therefore, to sustain sufficient SNR of the later Look‐Locker phases.

### 2.2 In vivo healthy volunteer study

The in vivo study was approved by the local institutional review board and all volunteers provided written informed consent before inclusion into this study. A total of 7 volunteers (male = 3, female = 4, mean age = 31.0 years [range, 19‐45 years]) without known cerebrovascular disease participated in this study.

All MR scans were performed on a Philips 3.0T Ingenia scanner (Philips, Best, The Netherlands) using a 32‐channel head coil. For presaturation of the static tissue, a WET module was used.^10^ Following the results of the Bloch equation simulations, the pCASL labeling duration and PLD were set to 800 ms and 200 ms, respectively. A multiphase Look‐Locker readout with a multishot 3D‐TFEPI sequence was used, in which 12 excitation pulses (including 2 start‐up dummy echoes) were applied per phase after each ASL preparation, which were filled in the feet‐head slice‐encoding direction of the k‐space. After each excitation pulse, 7 k‐lines were filled by EPI‐train (EPI‐factor of 7) in the right‐left phase‐encoding direction, and TR was set to 15 ms. A FA of 8° was used for the constant FA. Other imaging parameters were as follows: FOV = 220 × 176 mm, scan matrix = 176 × 176 reconstructed as 256 × 256 by zero‐filling, 70 slices with thickness of 1.3 mm were acquired and reconstructed as 140 slices of 0.65 mm, SENSE factor = 2.3 in right‐left direction and 1.2 in feet‐head direction. A total of 10 Look‐Locker phases were acquired with an interval of 180 ms, which resulted in the duration of a single Look‐Locker cycle (the interval between 2 WET presaturation modules) of 2800 ms.

**Figure 2:**
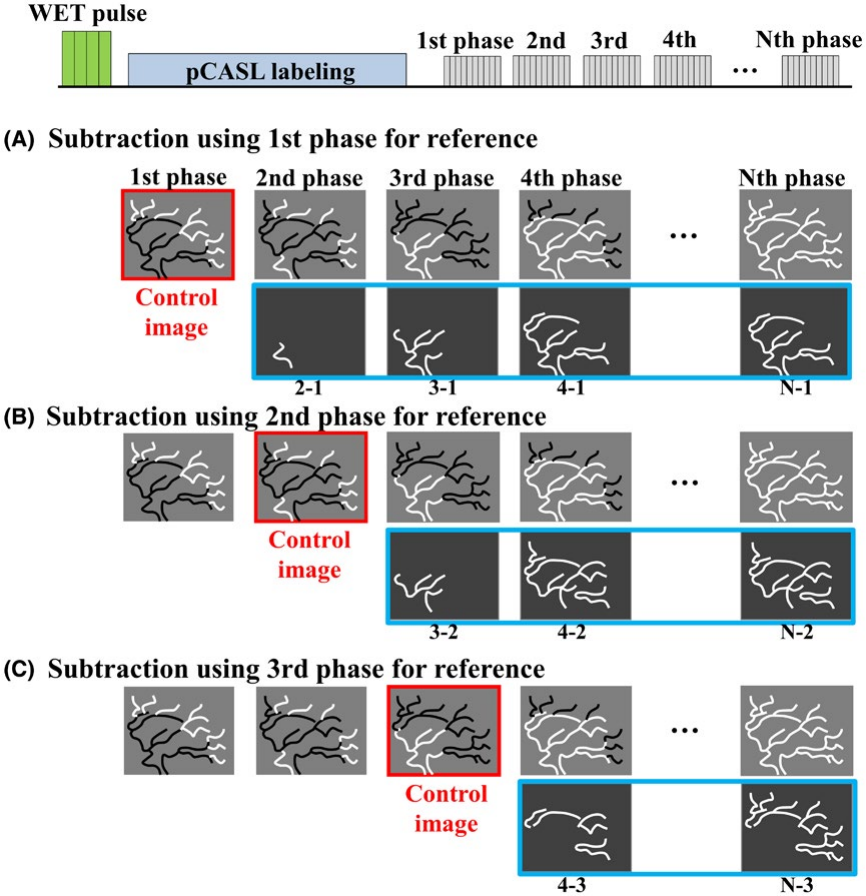
Basic sequence diagram of the pCASL‐ACTRESS approach and proposed subtraction schemes. In subtraction (A), (B), and (C), respectively, the 1st‐phase, 2nd‐phase, and 3rd‐phase are used as the control image for subtraction. Using later phases for the control image has 2 advantages: (i) slowly flowing labeled blood arriving later than the 1st‐phase will still be visualized correctly by using a later phase as control image, and (ii) subtraction using neighboring phases will minimize differences in the signal intensity of the background static tissue. The labeled and unlabeled blood are indicated in black and white, respectively

For all studies (described in the next paragraph), a conventional pCASL 4D‐MRA protocol was used, which allowed the generation of pCASL‐ACTRESS images as well as conventional pCASL 4D‐MRA images from the same data set, providing a reference for comparison. The total scan time was 5 min 19 s. The conventional pCASL 4D‐MRA was generated by a subtraction using both control and labeled images with the same PLD, which were acquired as separate Look‐Locker readout cycles. To generate the pCASL‐ACTRESS 4D‐MRA, only the labeled images were used and a new subtraction scheme was proposed to generate 4D‐MRA images (see Figure 2 and the “Extended inflow‐subtraction” section), thereby representing an effective scan time of 2 min 40 s.

The in vivo study consisted of 3 parts. Study‐(i): to confirm the stability of the static tissue signal over the Look‐Locker readout when using the above described parameters as optimized by Bloch equation simulations, nonselective pCASL 4D‐MRA images were acquired from 4 volunteers, and the signal intensity of the static tissue was measured by region of interest (ROI) analysis (see the “ROI analysis” section). Also, the pCASL‐ACTRESS images were generated from these data, and qualitative comparisons of the image quality were performed between pCASL‐ACTRESS and the conventional pCASL 4D‐MRA (see the “Extended inflow‐subtraction” and “Qualitative comparisons” sections ). Study‐(ii): vessel‐selective labeling of the right and left internal carotid artery (ICA) was performed in 4 volunteers (3 of these 4 volunteers were also part of the previous, nonselective study). Study‐(iii): separate visualization of arteries arising from the ICA and external carotid artery (ECA) was performed in 2 volunteers to demonstrate the clinical potential of this technique (e.g., visualization of bypass flow).

### 2.3 Vessel‐selective pCASL labeling

Very recently, we proposed an optimization of pCASL labeling parameters for vessel‐selective labeling that modify the shape of the spatial modulation function of vessel‐encoded pCASL to obtain (i) a sharp transition between the labeling and control conditions, and (ii) a broad and flat control region, to minimize the (partial) labeling of nontargeted arteries.^11^ In this study, one of the optimized settings in Suzuki et al^11^ with a maximum pCASL gradient strength (G_max_) of 6.0 mT/m and mean gradient strength (G_mean_) of 0.4 mT/m was used for selective labeling of the ICAs in Study‐(ii). However, for separate labeling of ICA and ECA in Study‐(iii), an even narrower labeling and a broader control condition was warranted and a G_max_ of 6.0 mT/m and G_mean_ of 0.2 mT/m were chosen. Other parameters for pCASL labeling were set as follows: pCASL labeling RF pulse duration of 0.5 ms, interval of 1.0 ms, and flip angle of 21°. The shapes of the employed spatial modulation functions as obtained by Bloch equation simulations are shown in Supporting Information Figure S1, which is available online.

### 2.4 Extended inflow‐subtraction

Unlike for PASL labeling which uses a very short duration of the labeling module (approximately 10‐20 ms), pCASL labeling relies on a much longer labeling module (800 ms in this study) and then labeled arterial blood will already have arrived in most of the arteries even when the first phase is acquired immediately after labeling has stopped. With a conventional subtraction approach between control and labeled images with identical PLD, only the outflow of labeled blood can be depicted, except for very slowly arriving arteries. To visualize the early inflow phase, “inflow‐subtraction” was proposed previously,^12^, ^13^ in which the 1st phase of the subtracted images is again subtracted by the subsequent phases. In ACTRESS approach, a similar inflow visualization is obtained by subtracting the 1st phase of the labeled images from the subsequent phases (Figure 2A, in which the labeled and unlabeled blood are illustrated in black and white, respectively). With this method, however, only the labeled blood that is already present in the 1st phase (illustrated in black in Figure 2A) can be visualized. This means that slowly flowing peripheral arteries in which labeled blood has not arrived yet in the 1st phase (illustrated in white in Figure 2A) will not be visualized even though labeled blood would have reached these distal segments in the 2nd or later phases. To avoid this problem, additional inflow‐subtractions were performed using the 2nd, 3rd, and even later phases as reference (control) images (Figure 2B,C). It should be noted that the labeled images exhibit inflow of nonlabeled blood (illustrated in white in Figure 2), whereas labeled blood exists in the control images (illustrated in black in Figure 2), which makes the subtraction order “labeled image – control image”.

### 2.5 ROI analysis

To assess the stability of the static tissue signal, the conventional subtraction (control minus labeled images with identical PLD) was performed using the pCASL 4D‐MRA data acquired in Study‐(i), and a temporal maximum intensity projection (MIP) across all phases (i.e., not the spatial direction) was produced for each slice. An ROI was manually drawn within the background static tissue on the 20th, 40th, 60th, 80th, 100th, and 120th slices avoiding obvious arteries and artifacts. These 6 ROIs were copied to the original nonsubtracted, non‐MIP images and the mean signal intensity was obtained as a function of PLD.

### 2.6 Qualitative comparisons

Spatial MIP images were generated in sagittal and transverse directions using the conventionally subtracted nonselective pCASL 4D‐MRA images as well as using the pCASL‐ACTRESS images as obtained by the extended inflow‐subtraction scheme described above. With these MIP images, qualitative comparisons between the conventional pCASL and pCASL‐ACTRESS were performed by a board‐certified neuroradiologist with 13 years of experience (N.F.) with respect to the existence of artifacts and/or major differences of vessel visualization as compared to the reference (conventional pCASL) images. Moreover, the depiction of the peripheral arteries was scored by means of a relative scoring system: 3 = same or very similar depiction as the reference, 2 = slightly poorer depiction, 1 = obviously poorer depiction.

Additionally, the maximum length of the occipital artery that was depicted on both pCASL‐ACTRESS and the conventional pCASL was measured. The occipital artery was chosen as a representative artery with slow flow. For this measurement MIPs with limited thickness of 50 mm (partial MIP) were generated in the coronal direction by centering on either the left or the right occipital artery to avoid over‐projection of other arteries.

## 3 RESULTS

**Figure 3:**
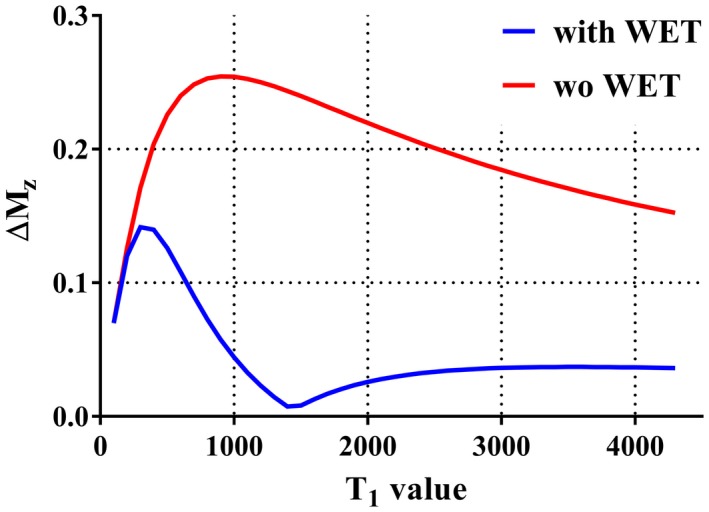
ΔM_z_ (the M_z_ variation defined as the difference between the maximum and minimum M_z_ over the Look‐Locker readout) obtained with variable T_1_ values ranged from 100 ms to 4300 ms in steps of 100 ms. ΔM_z_ was calculated from the simulated M_z_ during the readouts of 4th to 6th cycle, that is, removing the first 3 cycles to avoid the larger transition of M_z_ with long T_1_ value (e.g., CSF) before reaching the steady‐state (see Figure 1A)

Figure 1 shows the simulated longitudinal magnetization (M_z_) of GM, WM, and CSF over the labeling, PLD and Look‐Locker readout as obtained by Bloch equation simulations. With the parameters stated in the methods section for the in vivo study, the M_z_ variation (defined as the difference between the maximum and minimum M_z_ over the Look‐Locker readout: ΔM_z_) of GM, WM, and CSF were 0.023, 0.073 and 0.036 with a presaturation pulse (Figure 1A), which were lower than ΔM_z_ of 0.250, 0.253 and 0.152 obtained without applying a presaturation pulse (Figure 1B). Additionally, Figure 3 shows ΔM_z_ obtained over a range of T_1_ values from 100 ms to 4300 ms in steps of 100 ms, with and without a presaturation pulse. By applying a presaturation pulse, ΔM_z_ was kept lower than 0.1 for T_1_ value of 700 ms and longer. Also from the ROI analysis performed on the in vivo images, it was confirmed that the normalized variation of the mean signal intensity of static tissue was 8.98%.

**Figure 4:**
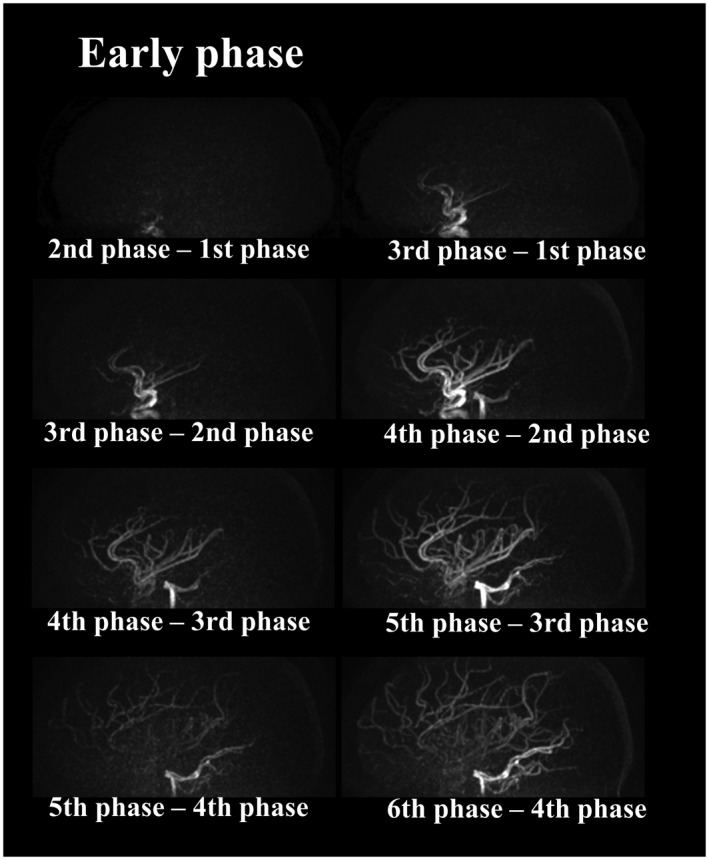
Representative images of the early inflow‐phase as obtained by the proposed subtraction schemes illustrated in Figure 2

**Figure 5:**
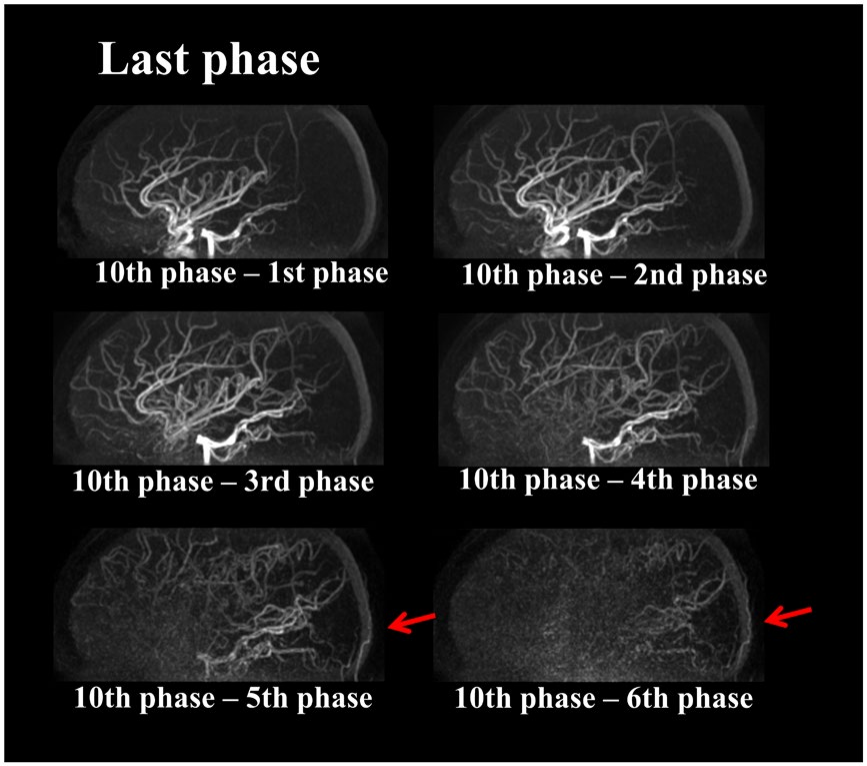
Representative images of the last peripheral‐phase as obtained by the proposed subtraction schemes illustrated in Figure 2. Slow blood flow in arteries such as the occipital artery are also well visualized (indicated by arrows)

**Figure 6:**
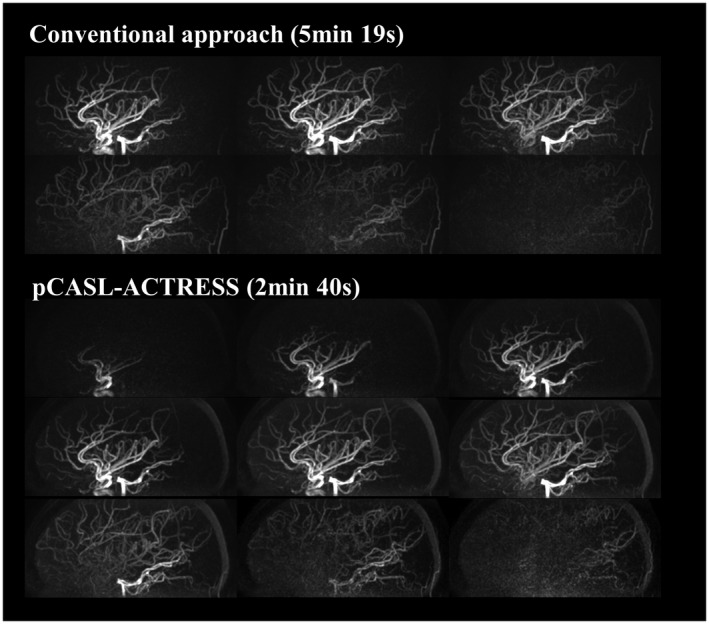
Comparison of images acquired by the pCASL‐ACTRESS approach (scan time of 2 min 40 s) and conventional approach (5 min 19 s)

**Figure 7:**
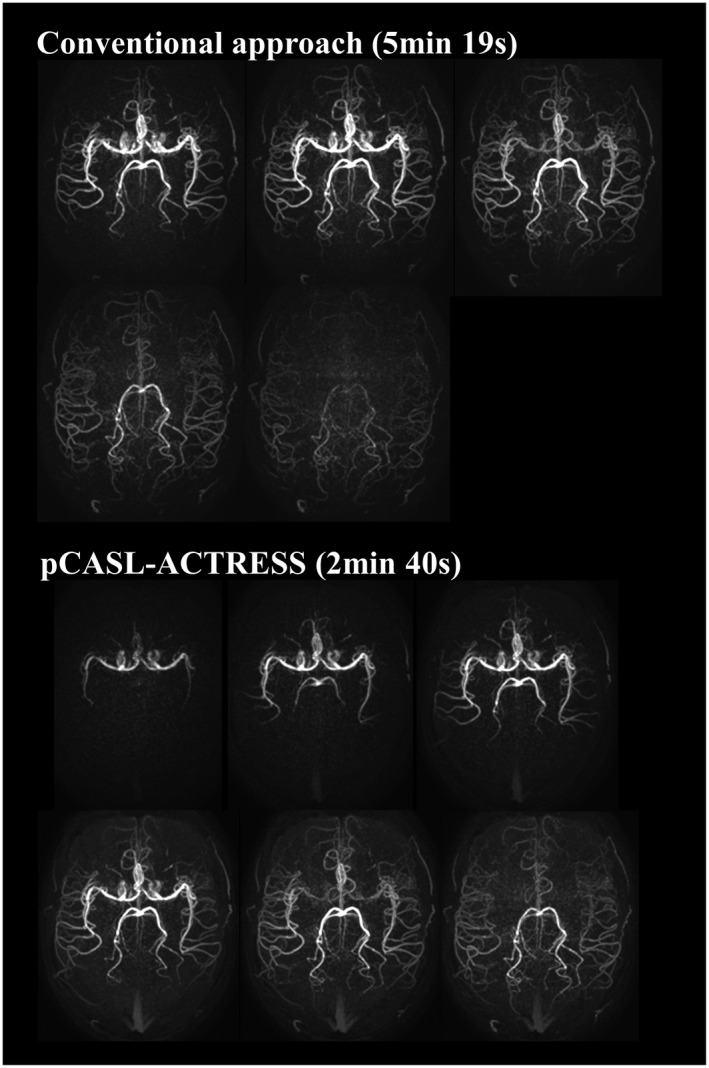
Comparison of images acquired by the pCASL‐ACTRESS approach (scan time of 2 min 40 s) and conventional approach (5 min 19 s)

**Figure 8:**
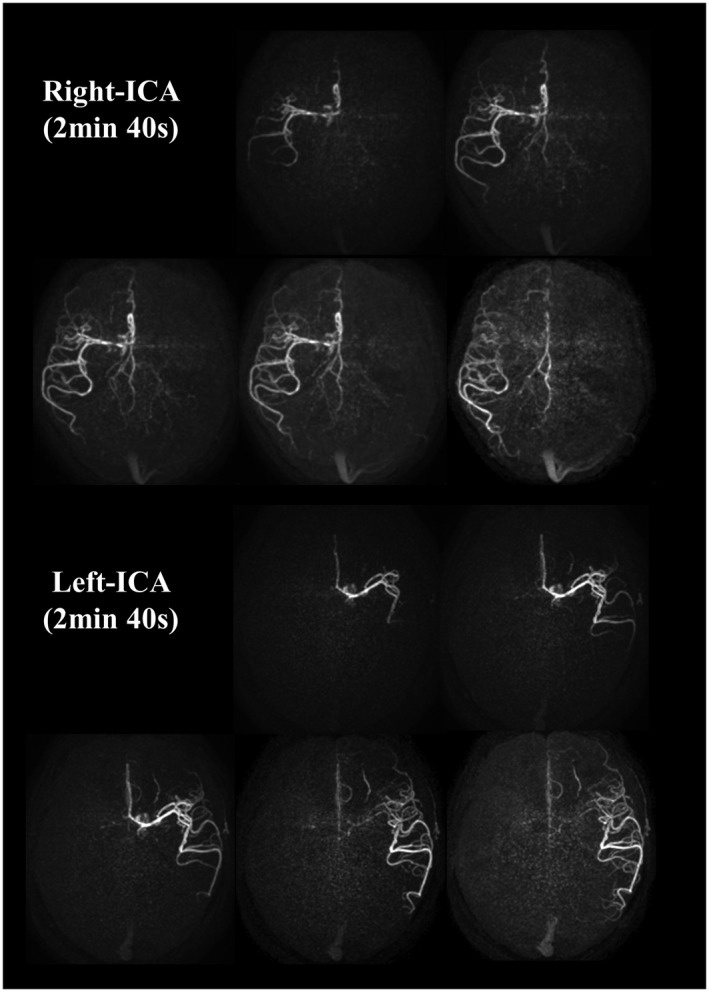
Representative images of vessel‐selective 4D‐MRA obtained by pCASL‐ACTRESS approach (scan time of 2 min 40 s per artery), in which either the right or left ICA was labeled

**Figure 9:**
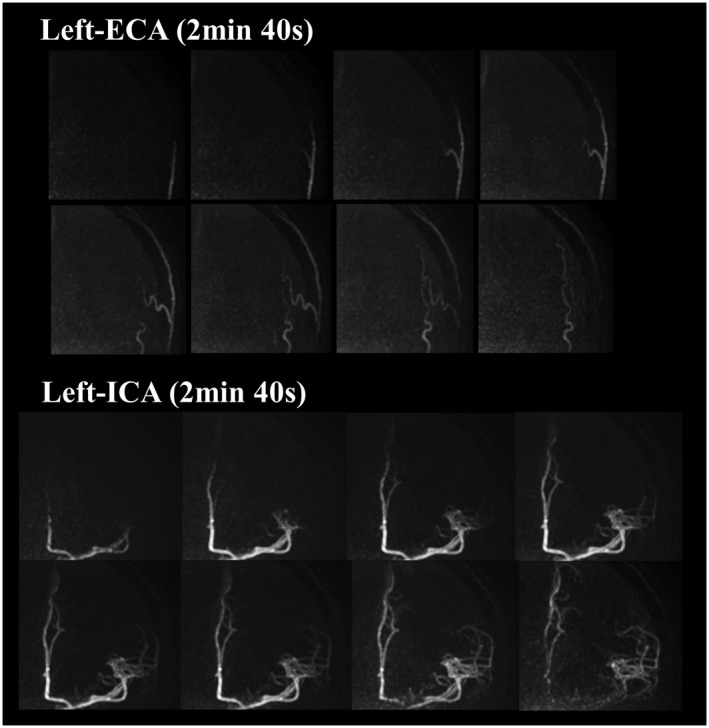
An example that illustrates the selective visualization of the ECA and ICA as obtained by the pCASL‐ACTRESS approach (scan time of 2 min 40 s per artery)

The representative images of the early inflow‐phases and the last peripheral‐phase as obtained by the extended inflow‐subtraction scheme are shown in Figures 4 and 5, respectively. By using a later phase as control image for subtraction, slowly arriving arterial blood such as those in the occipital artery were better visualized (indicated by arrows in Figure 5). A slight elevation of background signal was observed in pCASL‐ACTRESS as compared to the conventional approach, including signal within the superior sagittal sinus as indicated in the observer study (see next paragraph) (Figures 6 and 7). However, visualization of distal arteries was not hindered for any case. Figure 8 shows representative images of vessel‐selective pCASL‐ACTRESS 4D‐MRA from study‐(ii), in which the spatially selective labeling was performed targeting either the right or left ICA aiming at differentiating between these 2 vessels and the posterior circulation. However, this study‐(ii) resulted in ambiguous labeling for the ECA. Therefore, separate labeling between the ECA and ICA of the same hemisphere was attempted in 2 additional volunteers, from which 1 example is shown in Figure 9. These results show the potential of this approach for visualization of, e.g., a bypass artery.

In the observer study, it was found that the venous blood in the superior sagittal sinus was erroneously visualized in all pCASL‐ACTRESS 4D‐MRA, whereas none of the conventional pCASL depicted the superior sagittal sinus. We refer to the discussion for possible causes of this artifact. No other artifacts or major differences of vessel visualization were observed as compared to conventional pCASL 4D‐MRA. With regard to the depiction of peripheral arteries, all 4 images resulted in score = 3 (i.e., “same or very similar depiction as the reference”). As shown in Table 1, however, a 23.2% shorter depiction‐length of the occipital artery was found by pCASL‐ACTRESS as compared to the conventional pCASL 4D‐MRA. The PLD of images with the maximum depiction of occipital artery (which were used to measure the maximum length) ranged from 1100 to 1820 ms (mean value of 1302.5 ms).

**Table 1:** Maximum length of occipital artery measured on pCASL‐ACTRESS and the conventional pCASL

Subject	Target artery	ACTRESS (mm)	pCASL (mm)	Percentage (ACTRESS/pCASL)	PLD for maximum depiction (ms)
1	Right OA	41.82	50.20	83.3%	1100
	Left OA	26.49	38.16	69.4%	
2	Right OA	64.54	72.13	89.5%	1280
	Left OA	40.47	46.21	87.6%	
3	Right OA	28.11	39.50	71.2%	1280
	Left OA	53.2	74.22	71.7%	
4	Right OA	35.88	50.65	70.8%	1280
	Left OA	73.87	104.54	70.7%	1820

## 4 DISCUSSION

In this manuscript, we have presented a novel vessel‐selective 4D‐MRA acquisition which combines vessel‐selective pCASL with the ACTRESS acceleration approach.^3^ In the original ACTRESS approach based upon PASL for labeling, the application of vessel‐selective labeling was hampered by elevated background signal as a result of the disturbance of the equilibrium magnetization of the static tissue arising from the intersection of the labeling slab with the imaging slices. By using pCASL for labeling, in which the labeling pulses are applied approximately parallel to the imaging slices and, therefore, do not interfere with the magnetization in the imaging slices, the combination of vessel‐selective labeling and ACTRESS acceleration became possible. Although the long labeling module as used in pCASL sequence destroys the steady‐state of the magnetization by interrupting the continuous series of excitation pulses of the Look‐Locker TFEPI readout, thereby causing elevated background signal in the subtracted images, this could successfully be minimized by optimization of the relevant parameters, especially by using a presaturation pulse before the labeling module. The in vivo study proved that this approach allowed vessel‐specific 4D‐MRA in half of the scan‐time required for conventional ASL‐based acquisitions.

In this study, we also proposed the use of vessel‐selective pCASL with an optimized spatial labeling profile.^11^ Although it is a rectangular‐like inversion band, which has the spatial selectivity only in a single in‐plane direction, it enables a sharp transition between the labeling and control conditions as well as the optimized thin labeling band, thereby allowing enough freedom to label only a targeted artery. Moreover, broad and flat control condition is achieved without affecting the magnetization, which is essential to the ACTRESS approach. Super‐selective pCASL labeling based upon rotating in‐plane gradients achieves a circular labeling spot,^14^, ^15^ which would enable more focused labeling than the inversion band used in this study. However, residual influence on the magnetization outside of the target region was observed.^16^ This could produce partial saturation of the magnetization in untargeted arteries, which would in traditional pCASL 4D‐MRA not be a problem, because such an unwanted influence to the magnetization would be canceled out by means of the paired subtraction from the control image acquired with the same rotating gradients. When using the ACTRESS approach, however, such partial saturation will result in inclusion of the downstream vasculature in the generated 4D‐MRA, because the subtraction is performed between labeled images with a different PLD. Therefore, it is important to optimize the spatial modulation function of the longitudinal magnetization itself, and it is no longer sufficient to optimize the relative labeling efficiency, i.e., the difference between the labeling and control condition. A downside of this approach is that effective labeling is achieved in a rectangular region, which requires more careful planning to avoid inclusion of other arteries within the labeling plane.

In vessel‐encoded pCASL (ve‐pCASL) as proposed by Wong,^6^ vessel‐selective labeling is performed by a Hadamard‐encoding scheme, enabling the selective depiction of several arteries. For perfusion imaging, this Hadamard‐encoding can be performed as a replacement for averaging, and the territorial perfusion images can be obtained without time penalty. For 4D‐MRA acquisitions, however, all the scan time needs to be invested in the acquisition of the k‐lines needed for high spatial resolution, which leaves no scan time available to acquire multiple averages. Therefore, acquiring more Hadamard‐encodings for ve‐pCASL would directly result in a longer scan time for the complete 4D‐MRA acquisition. Time‐efficient acquisition of ve‐pCASL 4D‐MRA has been proposed previously,^11^ based upon 4 Hadamard‐encodings thereby separating 3 arteries (right ICA, left ICA and both vertebral arteries together), but this still results in a twice as long acquisition time as compared to a nonselective acquisition. Moreover, if separate visualization of the ECA or a bypass artery would be of interest, the number of Hadamard‐encodings would need to be increased even more, which again would result in an increase in scan time. By using the pCASL‐ACTRESS approach, selective visualization of a single target artery can be obtained in 2–3 min at a resolution of 1.25 × 1.25 × 1.3 mm, which can be, furthermore, flexibly applied only for a single or few arteries of interest, i.e., in a 1‐by‐1 manner.

With the original inflow‐subtraction approach,^12^, ^13^ peripheral arteries exhibiting slow flow in which the labeled blood will only arrive after the first readout would not be visualized. To avoid such missed visualization of peripheral arteries, a solution would be to increase the duration of the pCASL labeling ensuring that labeled blood has arrived in the complete arterial vasculature before the first phase is acquired. However, this would result in proportionally longer scan times and in a patient with slow flow, even longer label duration would need to be selected. In this study, subtraction was performed between all different combinations of PLDs (see Figure 1) to solve this issue. The proposed subtraction scheme was proven to also visualize arteries with late arrival, such as the occipital artery.

In the original ACTRESS approach using PASL, it is very important that the control image acquired in the first phase of the Look‐Locker readout does not contain the residual labeled blood from the previous Look‐Locker readout cycle.^3^ Such a carryover of the labeled blood to the next Look‐Locker cycle is unlikely to happen in pCASL‐ACTRESS, because the WET presaturation module applied before the pCASL labeling cancels the residual labeling remaining within the imaging volume. In this study, 4D‐MRA using the true pCASL‐ACTRESS approach was not acquired in the in vivo studies due to restricted examination time. Instead, pCASL‐ACTRESS 4D‐MRA was generated from the conventional pCASL data by using only the labeled Look‐Locker cycle. Considering above‐mentioned difference between the original ACTRESS using PASL and pCASL‐ACTRESS, however, such a time‐efficient way of comparison is presumed not to cause a biased estimation of the effect of the residual labeling. Moreover, our approach will minimize the influence of motion on the comparison, because both 4D‐MRA images are reconstructed from the same data.

There are several limitations that should be mentioned in this study. First, the parameter optimization was performed with the pCASL‐ACTRESS approach in mind, focusing on stabilization of the static tissue signal. Therefore, the optimized parameters might not be the best parameters for the conventional pCASL (non‐ACTRESS) images, e.g., FA of 10° could provide higher signal intensity within the arteries than the FA of 8° as used in this study. Second, the combination of parameters is not as flexible when using the ACTRESS approach as compared to a conventional pCASL‐4D‐MRA sequence. For example, when a conventional gradient echo sequence is used for the readout of pCASL‐ACTRESS instead of the proposed TFEPI sequence, the TR would be shortened and more excitations would be used after each ASL preparation to compensate for the absence of EPI (e.g., TR of 4 ms with 44 excitations for the same phase interval of 180 ms). This would result in a different equilibrium‐state, and lower FA and/or shorter pCASL labeling duration and PLD would be required to achieve the stability of the static tissue signal. Overall, this would result in lower SNR, and, therefore, the use of the TFEPI for pCASL‐ACTRESS was found to be highly preferable.

The third limitation is that we only tested our optimized settings in 7 healthy volunteers. In patient examinations, there could be pathology with shortened T_1_ values, such as a hemorrhage with methemoglobin present. As Figure 3 shows, M_z_ variation over the Look‐Locker readout would be larger for short T_1_ values, which could cause residual signal after subtraction. For clinical examination, therefore, further validation is required to study whether such signal from lesions would affect diagnosis. Lastly, an increase of the background signal was observed in pCASL‐ACTRESS, of which especially venous signal in the sagittal sinus was most noticeable. A plausible explanation for the enhanced venous signal would be the presence of slowly inflowing venous blood from outside of the imaging volume during the Look‐Locker readout. Such venous blood would experiences longer time to recover from the presaturation pulse and fewer excitation pulses, and, therefore, the M_z_ would be less saturated than other spins that have been staying within the imaging volume throughout the entire Look‐Locker readout. This difference introduces a gradual increase of M_z_ within the sagittal sinus over the Look‐Locker readout, thereby causing the enhanced visualization. We also attributed the weaker depiction of the occipital artery as found in the observer study to the slightly elevated background signal. However, as shown in the example images, these limitations would not impede many clinical applications.

## 5 CONCLUSIONS

In summary, we have presented an accelerated acquisition of vessel‐selective 4D‐MRA by combining vessel‐selective pCASL with the ACTRESS approach. By using vessel‐selective pCASL labeling, signal elevation of the background signal caused by intersection of the labeling slab and the imaging volume could be avoided. Application of a presaturation pulse and the use of optimized parameter settings minimized static tissue signal variations over the Look‐Locker readout, thereby minimizing subtraction errors. This approach allowed vessel‐specific visualization of the arterial vasculature in half of the scan‐time required for a conventional ASL‐based 4D‐MRA acquisition.

## Supporting information


**FIGURE S1** The shape of the spatial modulation employed in Study‐(ii) and Study‐(iii) as obtained by Bloch equation simulations. For Study‐(ii), maximum pCASL gradient strength (Gmax) of 6.0 mT/m and mean gradient strength (Gmean) of 0.4 mT/m were used. For Study‐(iii), Gmax of 6.0 mT/m and Gmean of 0.2 mT/m were used to obtain an even narrower labeling condition (and therefore a broader control condition). Other parameters for pCASL labeling were set as follows: pCASL labeling RF pulse duration of 0.5 ms, interval of 1.0 ms and flip angle of 21°
