# Supplementary material for: Acceleration of vessel‐selective dynamic MR Angiography by pseudocontinuous arterial spin labeling in combination with Acquisition of ConTRol and labEled images in the Same Shot (ACTRESS)
Source: Magn Reson Med. 2018 Dec 2;81(5):2995–3006. doi: 10.1002/mrm.27619 (PMC6492290; doi:10.1002/mrm.27619)
Supplement: Supplementary file 1 — FIGURE S1 The shape of the spatial modulation employed in Study‐(ii) and Study‐(iii) as obtained by Bloch equation simulations. For Study‐(ii), maximum pCASL gradient strength (Gmax) of 6.0 mT/m and mean gradient strength (Gmean) of 0.4 mT/m were used. For Study‐(iii), Gmax of 6.0 mT/m and Gmean of 0.2 mT/m were used to obtain an even narrower labeling condition (and therefore a broader control condition). Other parameters for pCASL labeling were set as follows: pCASL labeling RF pulse duration of 0.5 ms, interval of 1.0 ms and flip angle of 21° [file MRM-81-2995-s001.docx]

**Supporting information**

**Supporting Information Figure S1**


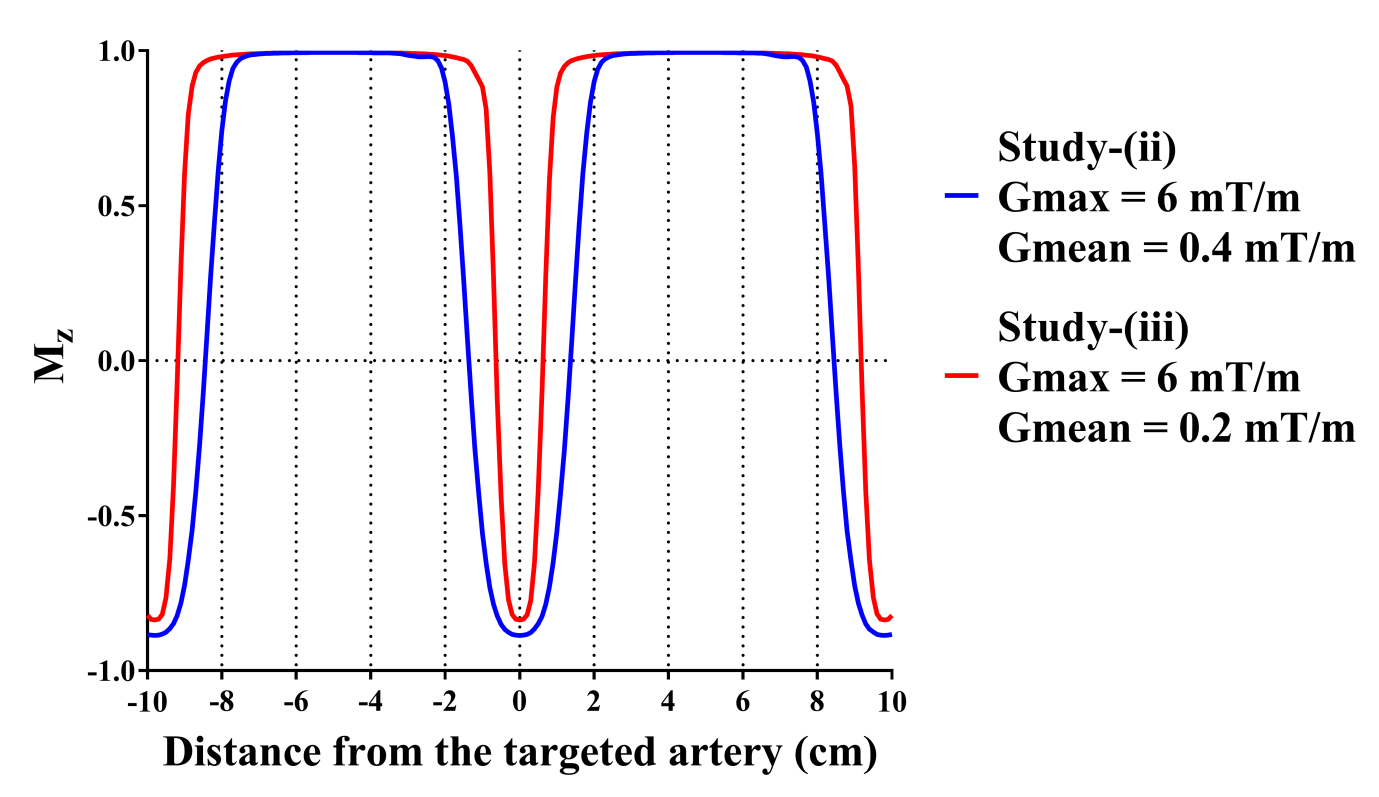


Supporting Information Figure S1: The shape of the spatial modulation employed in Study-(ii) and Study-(iii) as obtained by Bloch equation simulations. For Study-(ii), maximum pCASL gradient strength (G_max_) of 6.0 mT/m and mean gradient strength (G_mean_) of 0.4 mT/m were used. For Study-(iii), G_max_ of 6.0 mT/m and G_mean_ of 0.2 mT/m were used to obtain an even narrower labeling condition (and therefore a broader control condition). Other parameters for pCASL labeling were set as follows: pCASL labeling RF pulse duration of 0.5 ms, interval of 1.0 ms and flip angle of 21°.
